# Localization and Spectroscopic Analysis of the Cu(I) Binding Site in Wheat Metallothionein E_c_-1

**DOI:** 10.3390/ijms17030371

**Published:** 2016-03-11

**Authors:** Katsiaryna Tarasava, Jens Loebus, Eva Freisinger

**Affiliations:** Department of Chemistry, University of Zurich, 8057 Zurich, Switzerland; tarasava.katsiaryna@gmail.com (K.T.); jens.loebus@tosca-engineering.com (J.L.)

**Keywords:** metallothionein, wheat, copper ions, zinc ions, spectroscopy, luminescence spectroscopy

## Abstract

The early cysteine-labeled metallothionein (MT) from *Triticum aestivum* (common wheat), denoted E_c_-1, features two structurally well-defined domains, γ and β_E_, coordinating two and four Zn(II) ions, respectively. While the protein is currently assumed to function mainly in zinc homeostasis, a low amount of copper ions was also recently detected in a native E_c_-1 sample. To evaluate the observed copper binding in more detail, the recombinant Zn_6_E_c_-1 form was exposed to different amounts of Cu(I) ions and the resulting species characterized with spectroscopic methods. Data reveal that the first Cu(I) equivalent coordinates exclusively to the N-terminal γ-domain of the protein and replaces one Zn(II) ion. To analyze the ability of the γ-domain for coordination of monovalent metal ions in more detail, the γ-E_c_-1 peptide fragment was incubated with increasing amounts of Cu(I) and the process monitored with UV–VIS, circular dichroism, and luminescence spectroscopy. Closely similar spectra are observed regardless if the apo- or the metal ion-loaded and, hence, pre-folded forms, were used for the titration experiments with Cu(I). The results indicate that low amounts of Cu(I) ions displace the two metal ions subsequently and stoichiometrically, despite the different coordination geometry requirements of Cu(I) and Zn(II).

## 1. Introduction

Metallothioneins (MTs) are small proteins, typically consisting of up to roughly 90 amino acids, and are characterized by a high cysteine content of up to 30% [1,2]. This feature allows them to bind large numbers of metal ions with a preferentially d^10^ electron configuration, which are organized in metal-thiolate clusters. *In vivo*, MTs were shown to incorporate Zn(II), Cd(II), Cu(I), and Hg(II). This ability suggests a role of MTs in traffic and storage of essential metal ions, *i.e.*, mainly Zn(II) and Cu(I) [3,4] in the cell, as well as participation in the detoxification of harmful metal ions, such as Cd(II) and Hg(II) [5]. Free thiolate groups in the apo-forms of MTs were shown to undergo rapid oxidation under physiological conditions, leading to the proposal of a physiological role of MTs in the scavenging of reactive oxygen and nitrogen species [6].

**Scheme 1:**
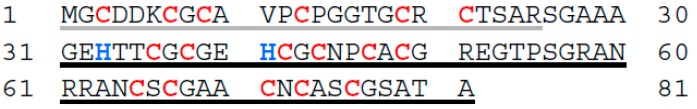
Amino acid sequence of wheat E_c_-1 including the N-terminal Met residue with Cys and His residues highlighted. Residues 2–25 belong to the γ-domain (gray underlined), residues 31–81 to the β_E_-domain (black underlined).

MTs are widely represented in all phyla of life showing a large diversity in their primary amino acid sequences, preference for metal ions, and cluster structures. According to the currently used classification system, all MTs are divided into 15 families based on sequence similarities and phylogenetic relationships [7]. The members of family 15, representing the plant MTs, are additionally separated into four subfamilies according to the observed cysteine pattern, *i.e.*, into the MT1, MT2, MT3, and E_c_ or early cysteine labeled proteins, sometimes also denoted as MT4 [8]. The latter protein subfamily is the focus of the current work, specifically the E_c_-1 MT of *Triticum aestivum* or common bread wheat. Wheat E_c_-1 was the first MT identified in plants and isolated from wheat germ as a Zn(II)-containing form [9]. The observed high Zn(II) content, as well as the mRNA transcription levels that are high in the developing and dry seed but decline rapidly after seed imbibition, suggest a role in intracellular regulation of zinc levels and zinc homeostasis during wheat embryo development [10]. E_c_-1 is composed of 81 amino acids and contains three consensus cysteine-rich regions separated by two cysteine free linkers (Scheme 1) [11,12].

The N-terminal cysteine-rich region (amino acids 2–25), also denoted as the γ-domain, hosts a M(II)_2_Cys_6_ cluster (PDB IDs 2L61, 2L62) [13]. The central and C-terminal cysteine-rich regions (amino acids 31–81) form the second and larger β_E_-domain, which encloses a Zn_3_Cys_9_ cluster, as well as a single ZnCys_2_His_2_ binding site that contains the two highly conserved histidine residues as ligands (PDB ID 2KAK) [14].

A more recent approach attempts to categorize MTs into either Zn- or Cu-thioneins based on their preference for and inducibility by mono- or divalent d^10^ metal ions [15]. While it was previously assumed that Cu-thioneins mainly occur in yeast, nowadays a role of plant MTs in Cu(I) metabolism including copper resistance, accumulation, and transport is also proposed based on the observed upregulation of certain MT transcripts in response to elevated copper concentrations [16,17,18,19] and growth studies using MT-deficient Arabidopsis plants [20,21]. However, the identification of the *in vivo* coordinated metal ions and hence the decision if a given MT is truly a Zn- or Cu-, or even a Cd-thionein or alike, is often difficult due to the challenges associated with protein isolation from native hosts.

Using the above definition, the E_c_-1 protein belongs to the Zn-thioneins as it was initially purified from wheat germs as a species containing five mol-equivalents of Zn(II) [9]. In addition, recombinant expression in *Escherichia coli* yields a Zn_6_E_c_-1 species that folds into a well-defined three-dimensional structure as shown by NMR spectroscopy, which further corroborates that Zn(II) is the native metal ion for this protein [13,14]. However, it is peculiar that the intracellular Zn(II) concentration is not correlated to the E_c_-1 mRNA levels in the wheat embryo, *i.e.*, transcription of E_c_-1 mRNA is not induced by Zn(II), but rather by the plant growth hormone abscisic acid [10].

The here presented study was triggered by a more recent publication, in which a sample of the average composition Zn_5.8±0.6_Cu_0.6±0.1_E_c_-1 was obtained from wheat germs [22]. While contamination of the sample with Cu(I) during purification cannot be completely excluded, a mixed metal species is in line with the initially-reported Zn_5_E_c_-1 species [9]. From the latter work it is not clear if the sample was analyzed for copper ions and, hence, on a hypothetical basis, additional Cu(I) binding might have been overlooked. Indeed, simultaneous binding of both Zn(II) and Cu(I) to MTs is a known phenomenon, especially in MTs that have been isolated from their native hosts [23,24,25]. In some instances, functional significance of additional Cu(I) binding was shown. For example, the brain-specific MT3 form, also denoted as growth inhibitory factor, as well as the human MT subtype MT-2a are able to prevent copper-mediated aggregation of the amyloid beta peptides Aβ_1–40_ and Aβ_1–42_ [26,27]. In the following we will present a number of spectroscopic and analytical analyses aimed to evaluate if low amounts of Cu(I) will be coordinated to a specific-site or randomly bound. This is especially interesting in view of the unique metal ion binding sites observed in the three-dimensional structure of E_c_-1: the complex arrangement, which also includes a Zn-finger like ZnCys_2_His_2_ binding site and a Zn_2_Cys_6_ cluster as known from certain yeast transcription factors [28], distinguishes E_c_-1 from the majority of other MTs that show Cys-only coordination of metal ions. The functional significance of this metal ion arrangement is presently unknown, but puts a question mark to a purely homeostatic function. Investigation of Cu(I) binding to E_c_-1 is performed with UV–VIS, circular dichroism, and luminescence spectroscopy, as well as limited proteolytic digestion to localize the bound Cu(I) in the γ- or β_E_-domain.

## 2. Results and Discussion

The E_c_-1 protein used in this study was recombinantly produced in *E. coli* without an additional purification tag and purified by precipitation and size exclusion chromatography. The purity of the obtained Zn_6_E_c_-1 form was confirmed by electrospray ionization mass spectrometry (ESI-MS, Figure S1), which shows signals for two species with and without the N-terminal methionine residue, a common feature of proteins expressed in *E. coli*.

### 2.1. Addition of One Equivalent of Cu(I) to Zn_6_E_c_-1

Due to the higher binding affinity of Cu(I) ions to thiolates, an excess of Cu(I) ions will presumably replace most, or even all, of the coordinated Zn(II) ions in Zn_6_E_c_-1. The question is, if the Cu(I) ions show any specificity for certain binding sites during this process. Therefore, and considering the Zn_5.8±0.6_Cu_0.6±0.1_E_c_-1 form isolated from wheat germ, we started our investigation with the characterization of a Zn(II)-E_c_-1 form containing up to one equivalent of Cu(I). For this, one equivalent of Cu(I) was added to a sample of Zn_6_E_c_-1 followed by the removal of free or weakly bound metal ions with either Chelex^®^ 100 resin, a chelator used for the removal of weakly bound transition metal ions, or by size exclusion chromatography (SEC). Subsequently, metal ion concentrations were determined with flame atomic absorption spectroscopy (F-AAS) and the protein concentration via quantification of thiol groups that are accessible for modification using the 2,2′-dithio-dipyridine (2-PDS) assay (Table S1) [29]. After Chelex^®^ 100 treatment 0.8 equivalents of copper and 5.3 equivalents of Zn(II) were coordinated to E_c_-1 indicating the replacement of approximately one Zn(II) ion by Cu(I). A very similar result is obtained after removal of unbound metal ions by SEC, *i.e.*, 0.7 equiv. of Cu(I) and 5.0 equiv. of Zn(II). These results strongly indicate that Cu(I) substitutes bound Zn(II) instead of occupying an additional binding site. MS spectra acquired at pH 2 show that no oxidation of Cys thiolate groups occurred and, in addition, residual binding of one equiv. of Cu(I) to the E_c_-1 protein can be observed (Figure S2).

To challenge the question of a potential Cu(I)-induced multimer formation, the hydrodynamic radii (r_H_) of Zn_6_E_c_-1 and CuZn_5_E_c_-1 were determined with dynamic light scattering (DLS) revealing identical values within the error limits, *i.e.*, 1.73 ± 0.11 and 1.63 ± 0.12 nm, respectively (Figure S3). Assuming a rod-like shape of the metal-loaded proteins this corresponds to a molecular mass of around 6.5 kDa (M_calc._ 7.7 kDa, see Experimental) and hence the observed r_H_ values are assigned to the monomeric proteins. No higher mass species are observed. Similarly, also the elution volumes of the two E_c_-1 forms observed with SEC are virtually the same.

### 2.2. Identification of the Cu(I) Binding Domain

**Figure 1:**
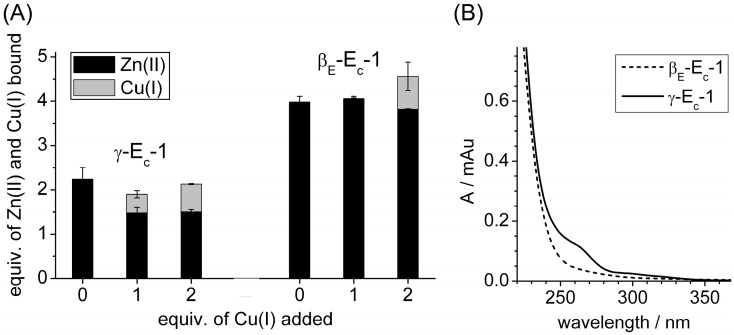
(**A**) Ratios of Zn(II) (black) and Cu(I) (gray) to protein domains after limited proteolytic digestion of Zn_6_E_c_-1 incubated with 0, 1, or 2 equiv. of Cu(I); (**B**) UV–VIS spectra of fractions from size exclusion chromatography (SEC) containing the γ- (solid line) or β_E_-E_c_-1 domain (dotted line) obtained after proteinase K cleavage of the Zn_6_E_c_-1 species which was incubated with 1 equiv. of Cu(I). The shoulder around 260 nm is assigned to the S → Cu(I) charge transfer transitions and only observed for the γ-E_c_-1 domain.

To identify the site of Cu(I) binding, *i.e.*, binding to the N-terminal γ- *versus* the C-terminal β_E_-domain, limited proteolytic digestion using proteinase K was performed, followed by separation of the fragments with SEC and determination of the metal ion concentrations with F-AAS as described before [12]. Three experiments with three replicates each were performed, *i.e.*, digestion of Zn_6_E_c_-1 and of the protein after addition of one or two equivalents of Cu(I). In all cases the SEC chromatograms show two peaks (Figure S4) assigned to the γ- and β_E_-E_c_-1 domains based on previous results [12] and on analysis with mass spectrometry (Figure S5). The Zn(II) and Cu(I) to protein fragment ratios for both fractions of all three protein samples are shown in Figure 1A.

In the absence of Cu(I) ions the β_E_-domain coordinates four and the γ-domain two Zn(II) ions as expected [11]. After addition of one equiv. of Cu(I), copper ions are exclusively detected in the fraction containing the γ-domain. This is supported by the UV spectra of the two SEC fractions (Figure 1B). While the fraction containing the β_E_-domain shows solely the typical features of a Zn(II)-binding MT, *i.e.*, a shoulder of the protein backbone transitions around 230 nm assigned to S → Zn(II) charge transfer bands, the UV spectrum of the γ-E_c_-1 containing fraction reveals in addition absorption features centered at 260 nm that are attributed to S → Cu(I) charge transfer transitions [30]. As observed, similarly, after addition of 1 equiv. Cu(I) to Zn_6_E_c_-1 (see above and Table S1) the Cu(I):protein ratio for the γ-domain fraction is also lower than 1:1, *i.e.*, only roughly 0.5:1. However, at the same time, 0.5 equiv. of Zn(II) were released and, thus, stoichiometric replacement of Zn(II) by Cu(I) took place suggesting that Cu(I) occupies the same or a very similar binding site as Zn(II). After addition of two equiv. of Cu(I) the binding specificity is lost and copper ions are now also detected in the SEC fraction containing the β_E_-domain.

### 2.3. Spectroscopic Studies of Cu(I) Binding to the Separate γ-E_c_-1 Domain

**Scheme 2:**
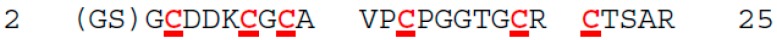
Amino acid sequence of the γ-E_c_-1 construct used in the study with Cys residues highlighted. The sequence is devoid of the N-terminal Met and contains two extra vector-derived GlySer residues (in parentheses, Materials and Methods). Numbering of residues corresponds to the one of the full-length sequence (see Scheme 1).

Stoichiometric replacement of a divalent by a monovalent metal ion is peculiar considering the different coordination modes observed for these metal ions in other MTs, *i.e.*, tetrahedral tetrathiolate coordination for Zn(II) and linear or trigonal coordination for Cu(I) [31,32]. To evaluate coordination of monovalent Cu(I) to the N-terminal γ-E_c_-1 domain in more detail, titrations were performed with the separate domain and monitored using UV, circular dichroism (CD), and luminescence spectroscopy. For this, a peptide with the sequence depicted in Scheme 2 was used, reflecting the amino acids 2–25 of the full-length protein.

Initial experiments were performed with the Zn_2_γ-E_c_-1 form as it has a defined three-dimensional structure and, hence, pre-formed metal ion binding sites [13]. Cu(I) ions were added stepwise to this form using a solution of the [Cu(CH_3_CN)_4_]^+^ complex in 1%–2% acetonitrile under strictly anaerobic conditions to avoid oxidation to Cu(II).

**Figure 2:**
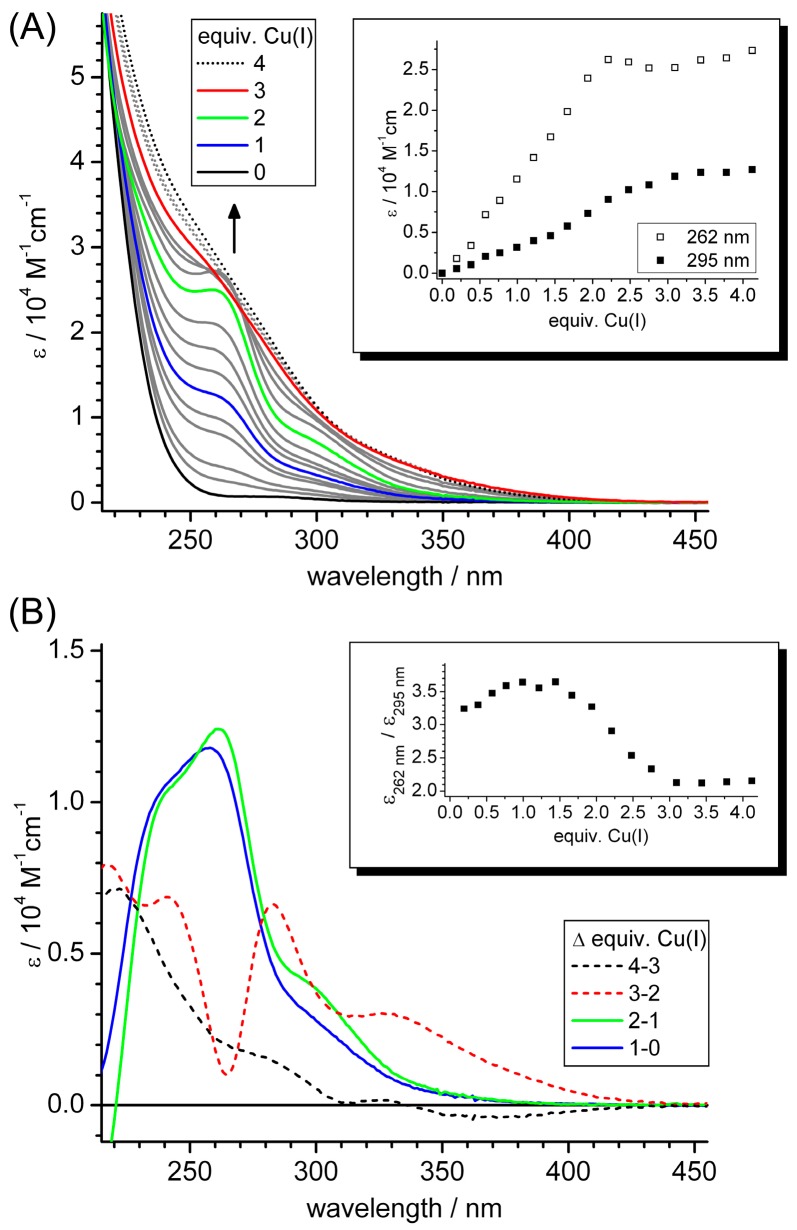
Analysis of the titration of Zn_2_γ-E_c_-1 with Cu(I) ions using UV–VIS spectroscopy. (**A**) Set of recorded. Dotted spectra indicate the point of constant spectral features. Inset: development of LMCT bands (262 nm) and cluster-centered transitions (295 nm); and (**B**) difference spectra (see text) of the titration revealing the spectral changes upon Cu(I) addition. Inset: absorptivity ratio ε_262 nm_/ε_295 nm_ to visualize the changing contribution of LMCT bands *versus* cluster-centered transitions.

Addition of Cu(I) ions leads to the development of a broad absorption envelope in the range between 240 and 400 nm dominated by two shoulders (Figure 2A). The prominent shoulder around 262 nm was previously assigned to S → Cu(I) charge transfer bands, while the less intense shoulder around 295 nm was related to cluster centered transitions [30]. These cluster centered transitions originate from d^10^–d^10^ overlap in a clustered structure with short Cu(I)–Cu(I) distances. The ligand to metal charge transfer (LMCT) bands centered at 262 nm show a linear increase up to the addition of approximately 2 equiv. of Cu(I) followed by a plateau. The intensity of these transitions is a direct measure for the number of Cys thiolate groups involved in Cu(I) coordination and, hence, allows an estimation of the number of ligands required for the binding of each metal ion [30,33]. Supposing that the maximum intensity at 262 nm reached upon addition of approximately 2 equiv. of Cu(I) results from the involvement of all six Cys thiolate groups of this domain in metal ion coordination, each thiolate ligand contributes approximately 4400 M^−1^·cm^−1^ to the overall molar absorptivity. Accordingly, coordination of one Cu(I) ion would require arithmetically 2.6, of two Cu(I) 6.0, and of three Cu(I) 5.8 thiolate ligands (Table 1). Involvement of, in round figures, three thiolate ligands in the coordination of the first Cu(I) ion is in agreement with a trigonal coordination sphere, as observed in the solid state structure of the *Saccharomyces cerevisiae* Cu_8_Cup1 metallothionein or in inorganic Cu(I)-thiolate model complexes [32,34]. Coordination of the second Cu(I) ion by another three thiolate ligands points to the formation of two independent metal ion binding sites with trigonal arrangements of terminally-coordinating Cys thiolate ligands each. As no additional thiolate ligands are available for the binding of the third Cu(I) ion, either transformation of originally terminal Cys thiolates to bridging ligands, recruitment of other non-Cys groups for coordination, or/and a structural rearrangement to (partial) linear dithiolate coordination must take place.

Upon addition of more than two equiv. of Cu(I) the absorption at 262 nm remains constant while it still increases in the lower energy region around 295 nm. These changes in the absorption envelope are best visualized in the difference absorption spectra, obtained by subtracting the UV spectrum of the preceding Cu(I) addition from the spectrum of the actual one. The difference spectra showing the spectral changes occurring from 0 → 1, 1 → 2, 2 → 3, and 3 → 4 equiv. of Cu(I) are depicted in Figure 2B. As mentioned above addition of the first two equiv. of Cu(I) leads to the development of a pronounced absorption band centered at 262 nm with shoulders around 236 and 300 nm. Coordination of the third equivalent is accompanied by a dramatic change: while the increase at 262 nm is nearly zero two well-resolved bands centered at 283 and 330 nm appear that fall in the range attributed to cluster centered transitions. The intensity of these transitions is a direct measure for the number of Cu(I) ions coordinated to the protein [33]. After addition of three equiv. of Cu(I) the spectral profile remains mostly constant. The changing contribution of LMCT and cluster-centered transitions, especially upon addition of 2–3 equiv. of Cu(I), is best demonstrated when plotting the ratio ε_262 nm_/ε_295 nm_ against the equiv. of Cu(I) ions added (Figure 2B, inset; the wavelength of 295 nm was selected for comparability with an analogous evaluation in the literature [30]). This ratio remains roughly constant up to the addition of approximately 1.5 equiv. of Cu(I) and it subsequently decreases up to 3 equivalents to remain constant thereafter. This feature, *i.e.*, an increase in absorptivity of the cluster centered transitions relative to the LMCT bands up to a certain amount of Cu(I) ions, results from a change in the bound Cu(I) to Cys stoichiometry and has been taken previously as an indication for the formation of, or the rearrangement to, a Cu(I)-MT complex with a defined Cu(I)-thiolate cluster structure [33]. A cluster consisting of three Cu(I) ions and six thiolate ligands is exemplified in the literature for an inorganic coordination compound, in which the three Cu(I) ions are arranged in a triangle bridged by three thiolate ligands [34]. The trigonal planar trithiolate coordination sphere is completed by one terminally coordinating thiolate ligand each per Cu(I) ion. Such an arrangement could, in theory, also satisfy the metal to ligand stoichiometry present in a Cu_3_γ-E_c_-1 species.

Analogous changes in band intensities are observed when instead of the Zn_2_γ-E_c_-1 species the Cd_2_- or apo-γ-E_c_-1 forms are titrated with Cu(I) ions (Figure S6). In both cases an increase of the absorptivity at 262 nm up to the addition of two equiv. of Cu(I) ions and a decrease of the ε_262 nm_/ε_295 nm_ ratio between roughly 1.5 and 3.0 equivalents is observed. The fact that identical results are obtained from the titration of apo-γ-E_c_-1 with Cu(I) suggests that the formation of the corresponding Cu(I)-γ-E_c_-1 complexes does not depend on the initial presence of a preformed cluster structure with divalent metal ions, *i.e.*, Zn_2_γ-E_c_-1 or Cd_2_γ-E_c_-1.

**Figure 3:**
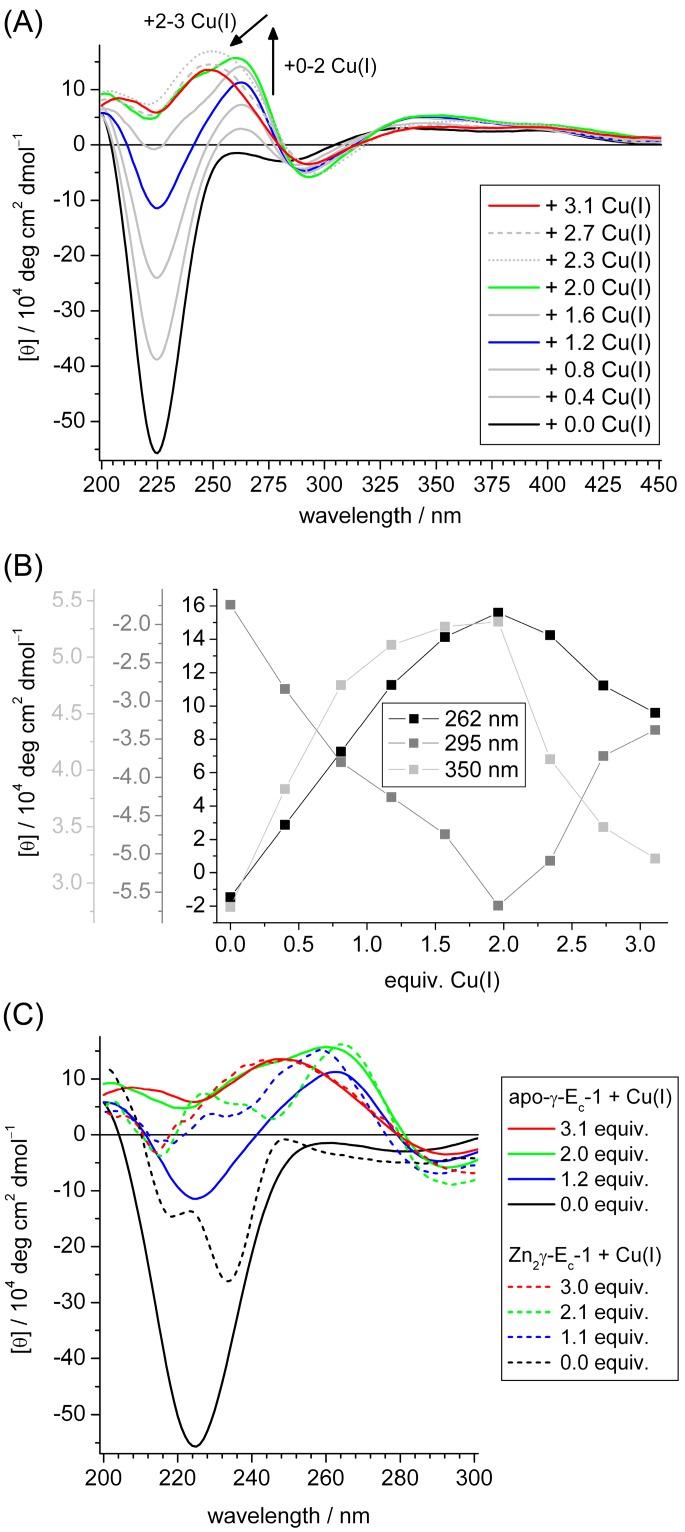
Titration of apo-γ-E_c_-1 with Cu(I) ions followed by CD spectroscopy. (**A**) Evolution of CD spectra; (**B**) course of ellipticity values at or close to the wavelength of the maxima observed in the CD spectra; and (**C**) comparison of selected spectra from (**A**) with the respective spectra obtained during the titration of Zn_2_γ-E_c_-1 with Cu(I) ions (see Figure S7).

To evaluate the structural changes induced by Cu(I) coordination further, titrations of apo-, Zn_2_-, and Cd_2_γ-E_c_-1 were also followed with CD spectroscopy. For the titration of apo-γ-E_c_-1 with Cu(I) evolution of extrema at (+)262 nm and (−)295 as well as in the range of (+)320–440 nm are observed (Figure 3A). These bands increase in ellipticity up to addition of two equiv. of Cu(I) and decrease, thereafter (Figure 3B). Addition of the third equiv. of Cu(I) causes a blue shift of the maximum at (+)262 nm to (+)247 nm and a broadening of the band. Hence, clearly, a structural rearrangement from the Cu_2_- to the Cu_3_-form takes place. For the titration of Zn_2_- and Cd_2_γ-E_c_-1 the spectra are more complex due to significant contributions of transitions, originating from the divalent metal ion-thiolate clusters, to the observed ellipticity profile, *i.e.*, up to approximately 275 nm for Zn_2_γ-E_c_-1 with extrema at (−)233.5 and (+)249 nm and up to approximately 300 nm for Cd_2_γ-E_c_-1 with extrema at (+)227, (−)241, (+)250, and (−)258 nm (Figures S7 and S8). Nevertheless, the ellipticity profiles of the species obtained after addition of two and three equiv. of Cu(I) to the Zn_2_- or apo-form are almost identical in the range above 260 nm (Figure 3C) indicating formation of closely-related species.

**Figure 4:**
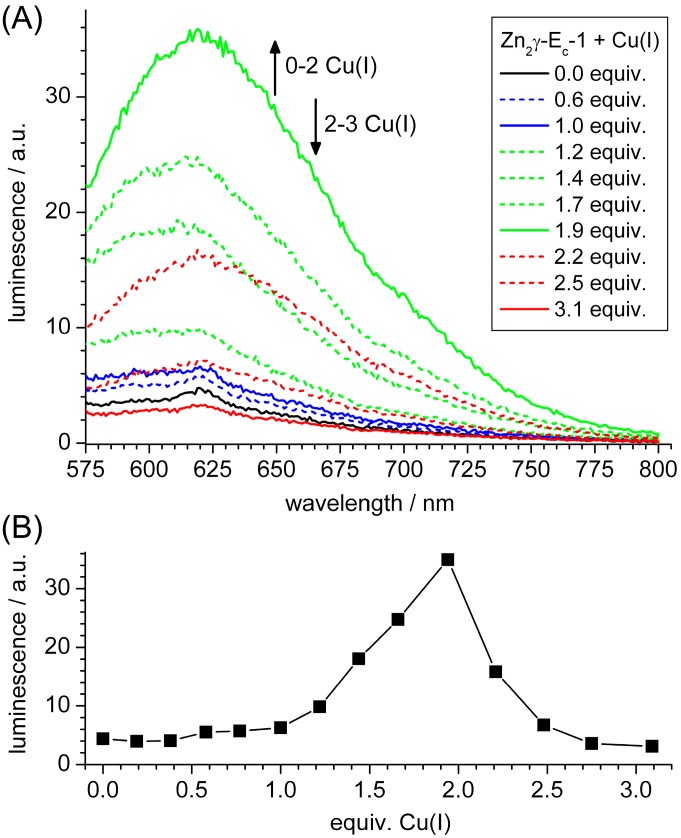
Dependence of room temperature luminescence at 600 nm after excitation at 280 nm from the amount of Cu(I) added to Zn_2_γ-E_c_-1. (**A**) Luminescence spectra; and (**B**) plot of luminescence at 600 nm against the equiv. of Cu(I) added.

In contrast to paramagnetic Cu(II), diamagnetic Cu(I) complexes exhibit a characteristic luminescence spectrum, which is often even observed at room temperature. The spectra recorded during the titration of apo- or Zn_2_γ-E_c_-1 with Cu(I) ions (Figure 4, Figure S9) show development of a broad band with highest intensity upon addition of two equiv. of Cu(I) followed by a decrease and complete disappearance with the addition of three equiv. of Cu(I). While the maximum of this band is initially found at 610 nm, a shift to 620 nm is observed upon addition of two or more Cu(I) ions. The position of these bands does not change with the chosen excitation wavelength, *i.e.*, 260, 280, or 340 nm. Noticeably, the increase of the band intensity is not proportional to the amount of Cu(I) ions added (Figure 4B): up to the addition of one equiv., only a minor increase in luminescence at 610 nm is observed, followed by a steep rise up to addition of two equiv. and a steep decrease to, again, a low luminescence value with further addition to approximately 2.5 equiv. of Cu(I).

A decrease of Cu(I)-thiolate luminescence can occur by solvent quenching due to insufficient shielding by the protein ligand [35]. Accordingly, the Cu(I)-thiolate coordination sites seem to be less shielded in the species obtained after addition of one and three equiv. compared to the species formed in the presence of two equiv. of Cu(I). As it can be assumed that the first Zn(II) ion to be replaced by Cu(I) is most likely the one that is easiest accessible, especially if the exchange is kinetically driven, a rather ineffective shielding of this first Cu(I) binding site by the protein ligand compared to the second site can be easily explained. Considering the titration of apo-γ-E_c_-1 with Cu(I), coordination of the first Cu(I) ion requires only three of the six free thiolate ligands. Accordingly, interchange between coordinating and free thiolate ligands can be expected, which will increase structural flexibility and, hence, the solvent exposure of the bound Cu(I) ion. The non-linear luminescence increase observed during the addition of 0–2 equiv. of Cu(I) to any of the γ-E_c_-1 forms indicates the non-cooperative formation of the Cu_2_-species. Hence, first a Cu_1_-species is formed followed either by a structural rearrangement to the luminescent Cu_2_-species with a shielded Cu(I)-thiolate core or, alternatively, the observed high luminescence might also solely result from coordination of the second Cu(I) ion in a highly-shielded binding site.

The decreased shielding against the solvent in the Cu_3_- *versus* the Cu_2_-species can be explained by two factors. On the one hand, a Cu_3_-thiolate cluster is larger and, hence, shielding by the protein ligand might be less efficient. On the other hand, or in addition, the increased Cu(I) to Cys ratio might impose a larger steric strain on the structure to enable Cu(I)-thiolate cluster formation, causing the structure to open up to the solvent.

Non-cooperativity of Cu(I) binding, at least for low Cu(I) to protein stoichiometries, can be also deduced for the full-length E_c_-1 protein. Luminescence spectra obtained before and after addition of one equiv. of Cu(I) to Zn_6_E_c_-1 are identical, *i.e.*, no increase of the room temperature luminescence at 610 nm is observed. Considering that the first equiv. of Cu(I) is exclusively bound to the γ-domain (Figure 1), this result strongly indicates the specific formation of a CuZn_5_-E_c_-1 form and the absence of any Cu_2_Zn_4_E_c_-1 species with two Cu(I) ions bound to the γ-domain as the latter would show strong luminescence. This is significant to note as, e.g., a 1:1 mixture of Cu_2_Zn_4_ and Zn_6_-species cannot be differentiated from a pure CuZn_5_-species solely based on the determination of metal ion-to-protein ratios.

### 2.4. Metal Ion to Protein Stoichiometries of γ-E_c_-1 Species Formed during the Titrations with Cu(I) Ions

**Figure 5:**
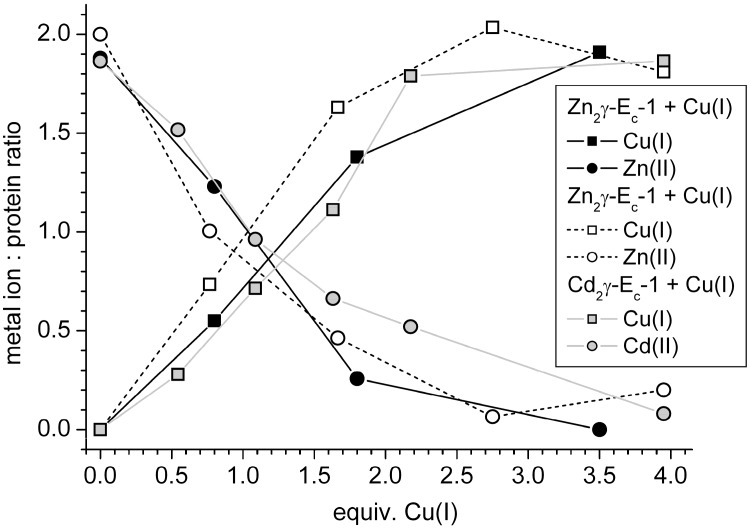
Metal ion composition of γ-E_c_-1 species obtained during the titration of Zn_2_- (two independent experiments) or Cd_2_E_c_-1 with increasing amounts of Cu(I) ions. Loosely-bound metal ions were removed with Chelex^®^ 100 resin, and metal ion concentrations determined with F-AAS.

Mass spectrometry, as a method to obtain information about the different species formed during the titrations, was not applicable as the quality of the spectra did not allow a clear differentiation between Zn(II) and Cu(I) ions due to the similarity of their masses. A possibility might be the acquisition of spectra at a pH value low enough to release all Zn(II) ions from the Cys thiolate ligands, *i.e.*, around pH 3.5 for Zn_2_ γ-E_c_-1 [36], but high enough to still enable Cu(I) binding, *i.e.*, above pH 3.0 as shown for Cu(I)-substituted human MT-3 and a MT form from *Candida albicans* [37,38]. Nevertheless and in addition, mass spectroscopy usually does not permit a quantitative analysis of the species, in particular when differently charged forms are to be analyzed. Hence, only bulk metal ion-to-protein stoichiometries were determined here using analytical methods. For this, samples were taken after each addition of Cu(I) ions to Zn_2_- or Cd_2_γ-E_c_-1, incubated with Chelex^®^ 100 resin to remove loosely bound metal ions, and the respective metal ion content was quantified with F-AAS. The plot representing the metal ion composition of the species is depicted in Figure 5. The data show that the coordinated Zn(II) or Cd(II) ions are replaced stoichiometrically by Cu(I) ions. Additionally, a maximum ratio of two Cu(I) ions per peptide is observed, even after addition of up to four equivalents of Cu(I). Accordingly, binding of the third Cu(I) equivalent, that was observed in the UV, CD, and luminescence spectra, occurs with rather low affinity as this Cu(I) can be removed by Chelex^®^ 100.

### 2.5. Biologically Relevant or an Artifact?

Clearly the question, if there is any biological role for Cu(I) binding to wheat E_c_-1, is impossible to answer with the current knowledge. The expression of E_c_ or MT4 related genes, and also the production of the encoded proteins in response to Cu(I) ions, was never reported so far; however, it is known for a number of other MTs. For example, when protein extracts from embryos and shoots of rice seeds were germinated for six days in 200 μM Cu(II) solution a five-fold increase of MT2 production was observed compared to seedlings that were not exposed to excess Cu(II) [39]. However, no protein-to-metal contents were determined in this study and hence it is unknown if the MT2 form actually contained copper ions. A correlation between E_c_ protein production *in vivo* and Cu(I) ions was so far only suggested by yeast complementation assays, *i.e.*, both *A. thaliana* E_c_ forms are able to complement the function of the yeast Cup1 MT leading to greater survival and higher accumulation of copper in the cell [20].

Nevertheless, despite the current assignment of wheat E_c_-1 as a typical Zn-thionein, it has to be noted that the fully metal ion-loaded Zn_6_E_c_-1 form has never been isolated from its native *in vivo* source so far. Therefore, the possibility of an alternative Cu(I)-containing species occurring *in vivo* cannot be completely ruled out, especially not in light of the results obtained here that show that the Cu(I) ion is not bound adventitiously to the protein, but rather specifically to Cys residues accompanied by replacement of a coordinated Zn(II).

## 3. Materials and Methods

### 3.1. Chemicals and Solutions

Tris and IPTG was obtained from Biosolve (Valkenswaard, Netherlands), Chelex^®^ 100 resin from Bio-Rad (Cressier, Switzerland), Luria-Bertani (LB) media used for *E. coli* expression from Roth AG (Arlesheim, Switzerland), and *Tritirachium album* proteinase K from Qbiogene (Lucerna Chem AG, Luzern, Switzerland). All other chemicals, including metal salts, were purchased in ACS grade from Sigma-Aldrich (Buchs, Switzerland) and Fluka (Buchs, Switzerland). All solutions were degassed under vacuum, followed by saturation with nitrogen. When complete absence of oxygen was required, solutions were degassed by three freeze-thawing cycles on a vacuum line, and subsequently saturated with argon.

### 3.2. Protein Expression and Purification

Purification of Zn_6_E_c_-1. For the expression of full-length E_c_-1 a construct that was cloned into the pTYB2 vector (New England Biolabs, USA) was used. Insertion of a STOP codon at the protein C-terminus just prior to the C-terminal intein purification tag allows tag-less expression [11]. *E. coli* BL21 (DE3) cells were grown in LB medium and expression was induced at an OD value at 600 nm of 0.6 by addition of isopropyl β-d-1-thiogalactopyranoside (IPTG) to a final concentration of 1 mM in presence of 1 mM ZnCl_2_. After 5 h at 30 °C, cells were collected by centrifugation at 6000 *g* for 10 min at 4 °C. Cell lysis was performed by sonication in 1× phosphate-buffered saline (PBS), pH 8.6, containing 100 mM dithiothreitol (DTT). The cleared cell lysate obtained by centrifugation (30,000 *g*, 60 min, 4 °C) was titrated drop-wise and under stirring with a 10% (*w*/*w*) solution of trifluoroacetic acid (TFA) up to a final concentration of 0.75% (*w*/*w*) TFA. The formed white precipitate was removed by centrifugation (13,200 *g*, 2 min, 4 °C), and the supernatant was, again, treated drop-wise with 100% (*w*/*w*) TFA under rigorous stirring up to a final concentration of 4% (*w*/*w*) TFA. The mixture was centrifuged again (13,200 *g*, 2 min, 4 °C) and the resulting solution mixed with a ZnCl_2_ solution to a final concentration of 10 mM. The pH was raised to pH 3 with a 10 M NaOH solution, Tris base was added to a final concentration of 100 mM and, subsequently, again a 10 M NaOH solution was used to increase the pH to 8–9, accompanied by a color change of the previously colorless solution to reddish brown and the formation of a small amount of a brownish precipitate. After centrifugation (13,200 *g*, 10 min, 4 °C) the supernatant was dialyzed twice against 1 mM Tris-HCl, 1 mM ZnCl_2_, pH 8, using SnakeSkin^®^ dialysis tubing (MWCO 3.5 kDa, Perbio Science, Ecublens, Switzerland) and further purified by size exclusion chromatography with a HiLoad 16/600 Superdex 75 pg column (GE Healthcare, Little Chalfont, UK) equilibrated in 10 mM Tris-HCl, 10 mM NaCl, pH 8. Zn_6_E_c_-1 elutes as a monomer around 75 mL. Final protein yields are around 2 mg per liter of cell culture.

The γ-E_c_-1 domain was recombinantly expressed as a glutathione *S*-transferase (GST) fusion protein using the pGEX-4T vector and purified as previously described [13]. To promote complete cleavage of the GST tag by thrombin, two additional Gly and Ser residues were added to the N-terminus of the γ-E_c_-1 domain.

### 3.3. Proteinase K Cleavage

Typically, 0.5 mL of a 60 μM Zn_6_E_c_-1 solution was incubated with proteinase K using a molar ratio of 50:1 in 50 mM Tris-HCl, 10 mM CaCl_2_, pH 8.0, at room temperature for 40 min without mixing. The digestion mixture was immediately applied to a Superdex Peptide 10/300 GL column (GE Healthcare) that was pre-equilibrated with 10 mM ammonium acetate, pH 7.5. Fractions containing cleaved protein fragments were collected for further analysis. For the analysis of Cu(I) binding, the corresponding amount of Cu(I) (see below) was added prior to proteolytic cleavage and 1% acetonitrile was added to the column buffer. Protein concentrations were determined by thiol group quantification with the 2-PDS assay assuming only five accessible Cys for the γ-domain after addition of 1 or 2 equiv. of Cu(I) based on control experiments and 10 accessible Cys for the fraction containing the β_E_-domain after addition of 2 equiv. of Cu(I) (see also Table S1) [29]. Metal ion concentrations were determined with F-AAS in 0.2 M HNO_3_ using an AA240FS spectrometer (Agilent Technologies AG, Basel, Switzerland).

### 3.4. Titration of γ-E_c_-1 with Cu(I)

All titration experiments were performed using a septum sealed cuvette, which was filled under strictly anaerobic conditions within a glove box equipped with a palladium catalyst and filled with a 5% hydrogen/95% nitrogen gas mix (Coy Lab, Grass Lake, MI, USA). A 1 mM Cu(I) solution was prepared from [Cu(MeCN)_4_](BF_4_), which was synthesized according to the literature [40], and 2% (*v*/*v*) acetonitrile was added to stabilize the Cu(I) oxidation state. The exact concentration was determined by F-AAS and the solution further diluted prior to the experiments so that 4 µL correspond to 1 equiv. of Cu(I). This solution was transferred into a micro syringe (Hamilton, Bonaduz, Switzerland) sealed with paraffin and used to titrate 20 μM solutions of apo-, Zn_2_-, or Cd_2_γ-E_c_-1 in 10 mM Tris-HCl, 10 mM NaCl, 2% acetonitrile, pH 7.5. At the end of each titration series the actual metal ion content in the cuvette was again assessed by F-AAS to exclude experimental errors.

### 3.5. CD Spectroscopy

All CD spectra were measured in 10 mM Tris-HCl, 10 mM NaCl, pH 7.5, on a J-715 spectropolarimeter (Jasco, Japan) at 25 °C in the range of 200–500 nm with a scanning speed of 50 nm·min^−1^ using three acquisitions per spectrum.

### 3.6. Mass Spectrometry

Samples were prepared in 10 mM ammonium acetate, pH 7.5, in 50% (*V*/*V*) acetonitrile and injected directly, or after acidification with HCl to pH 2 for analysis of the apo-forms, in a quadrupole time-of flight (TOF) Ultima API spectrometer (Waters, Elstree, UK). Scans were accumulated and further processed with the software MassLynx 3.5 (Waters, Elstree, UK). Deconvolution of mass spectra was done by applying the maximum entropy algorithm of the MassLynx tool MaxEnt1. Electrospray parameters were capillary voltage 2.8 V and cone voltage 60 V with a source temperature of 80 °C.

### 3.7. Luminescence Spectroscopy

Luminescence spectra in the range of 300–800 nm were collected at 293 K on a Cary Eclipse Fluorescence Spectrophotometer (Varian AG, Zug, Switzerland) using medium mode for the PMT detector voltage (600 V), an excitation slit width of 5 nm, a scan speed of 600 nm·min^−1^, and the three excitation wavelengths 260, 280, and 300 nm, corresponding to the observed bands in the absorption spectra. No smoothing function and post-acquisition correction was applied.

### 3.8. DLS Measurements

DLS measurements were performed with a DynaPro Titan instrument equipped with a temperature controlled micro sampler (Wyatt Technology Corporation, Santa Barbara, CA, USA). The measurements were performed using 50 µL of 50 µM samples at 15 °C in a buffer containing 10 mM Tris-HCl, 10 mM NaCl, 2% acetonitrile, pH 7.5, with 10 data acquisitions per measurement. Protein masses were calculated based on shape models implemented in the Dynamics^®^ software (Wyatt Technology Corporation, Santa Barbara, CA, USA).

## 4. Conclusions

In view of the Zn_5.8±0.6_Cu_0.6±0.1_E_c_-1 species obtained from wheat germ [22], the coordination of Cu(I) ions to different E_c_-1 forms was investigated in more detail providing the following key results. (i) The first equivalent of Cu(I) is specifically bound to the N-terminal γ-E_c_-1 domain with concurrent replacement of one Zn(II) ion. This Cu(I) ion is coordinated by three thiolate ligands; (ii) coordination of a second equiv. of Cu(I) is less domain-specific and accompanied by an increase of the Cu(I) content of both the γ- and the β_E_-domain; additional studies were conducted with the isolated γ-E_c_-1 domain showing (iii) that addition of a second equiv. of Cu(I) leads to the formation of a Cu_2_ γ-E_c_-1 species without residual Zn(II) ions and that all thiolate ligands are involved in Cu(I) coordination. The minor contribution of cluster centered transitions to the absorption spectra suggests a long Cu(I)-Cu(I) distance and consequently the absence of bridging thiolate ligands [30,33]; (iv) with three equiv. of Cu(I) a Cu_3_γ-E_c_-1 species is observed showing cluster centered transitions both in the UV–VIS and CD spectra corroborating some sort of cluster formation. Nevertheless, the binding affinity for the third Cu(I) ion is considerably lower and the metal ion can be removed by incubation with Chelex^®^ 100 resin; (v) the two binding sites in Cu_2_γ-E_c_-1 are occupied sequentially and not cooperatively, as revealed by luminescence spectroscopy; and (vi) luminescence spectra of the full-length protein together with data obtained from limited proteolytic digestion experiments strongly indicate the formation of a CuZn_5_E_c_-1 species after addition of one equiv. of Cu(I) opposed to a mixture of, e.g., Zn_6_E_c_-1 and Cu_2_Zn_4_E_c_-1 species with two Cu(I) ions coordinated to theγ-E_c_-1 domain.

The results gained in this study should be considered as a direction for further studies *in vivo*. Analysis of the metal ion composition and alteration of E_c_-1 concentration on the proteomic and transcriptional level under copper stress conditions will provide valuable insights into the role of plant MTs in response to heavy metal toxicity.

## Figures and Tables

**Table 1:** Molar absorptivity values after addition of the specified amounts of Cu(I) ions to Zn_2_γ-E_c_-1 and arithmetic numbers for the amount of terminal Cys thiolate ligands involved in Cu(I) coordination at each point.

	+1 Cu(I)	+2 Cu(I)	+3 Cu(I)
ε_262 nm*,*_M^−1^·cm^−1^	11,521	26,208	25,226
number −S^−^	2.6	6.0	5.8

## Supplementary Materials

Supplementary materials can be found at http://www.mdpi.com/1422-0067/17/3/371/s1.

## Abbreviations

The following abbreviations are used in this manuscript:

2-PDS	2,2′-dithio-dipyridine	F-AAS	Flame atomic absorption spectroscopy
CD	Circular dichroism	LMCT	Ligand-to-metal charge transfer
DLS	Dynamic light scattering	MT	Metallothionein
ESI-MS	Electrospray ionization mass spectrometry	SEC	Size exclusion chromatography
