# Supplementary material for: Localization and Spectroscopic Analysis of the Cu(I) Binding Site in Wheat Metallothionein Ec-1
Source: Int J Mol Sci. 2016 Mar 11;17(3):371. doi: 10.3390/ijms17030371 (PMC4813231; doi:10.3390/ijms17030371)
Supplement: Supplementary file 1 [file ijms-17-00371-s001.pdf]

Supplementary Materials: Localization and Spectroscopic Analysis of the Cu(I) Binding Site in Wheat Metallothionein E_c-1

Katsiaryna Tarasava, Jens Loebus and Eva Freisinger

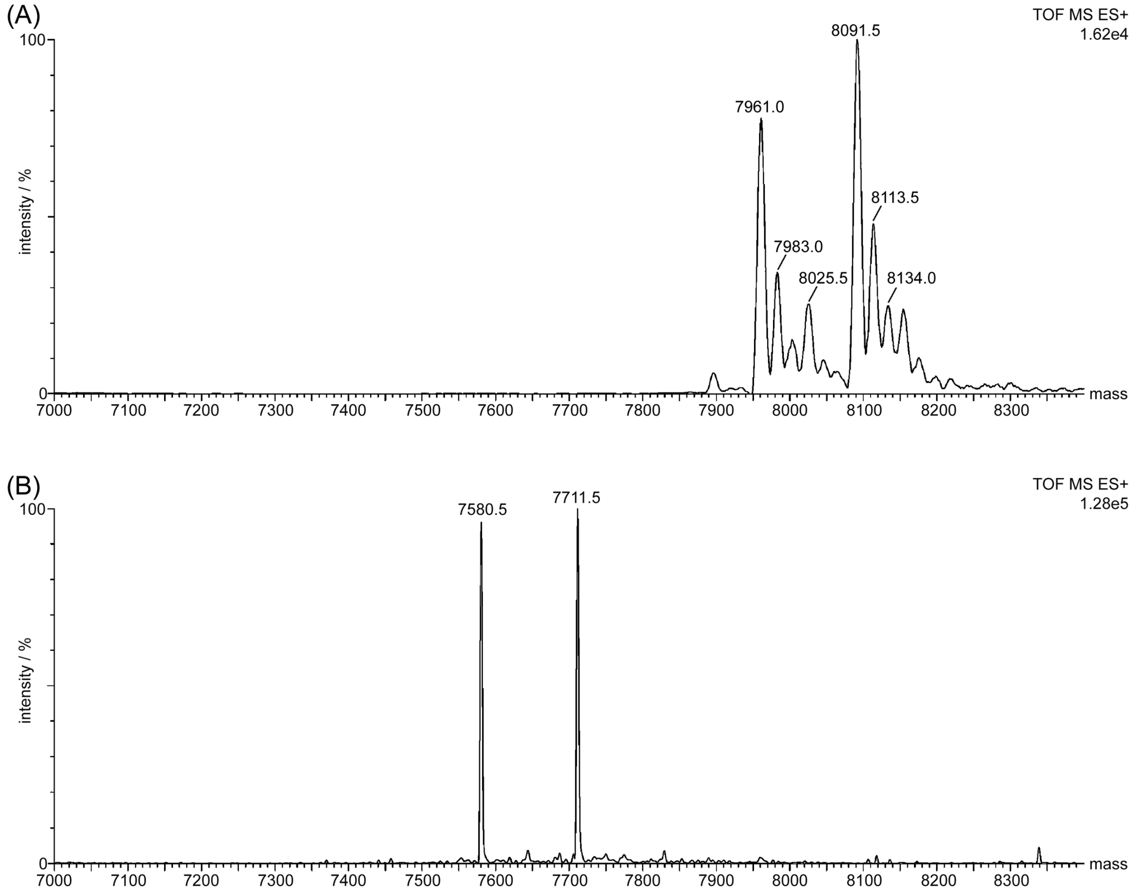

Figure S1. Deconvoluted ESI-MS spectra of Zn_6E_c-1 acquired at (**A**) pH 7.5 and (**B**) pH 2, representing the fully Zn(II)-loaded and apo species, respectively. The two main species in each spectrum correspond to the full-length protein with and without the N-terminal Met residue. Minor signals in (**A**) are due to Na^+-adduct formation. M(calc): Zn_6E_c-1 8092.8 Da, Zn_6E_c-1 (−Met) 7961.7 Da, apo-E_c-1 7712.5 Da, apo-E_c-1 (−Met) 7581.4 Da.

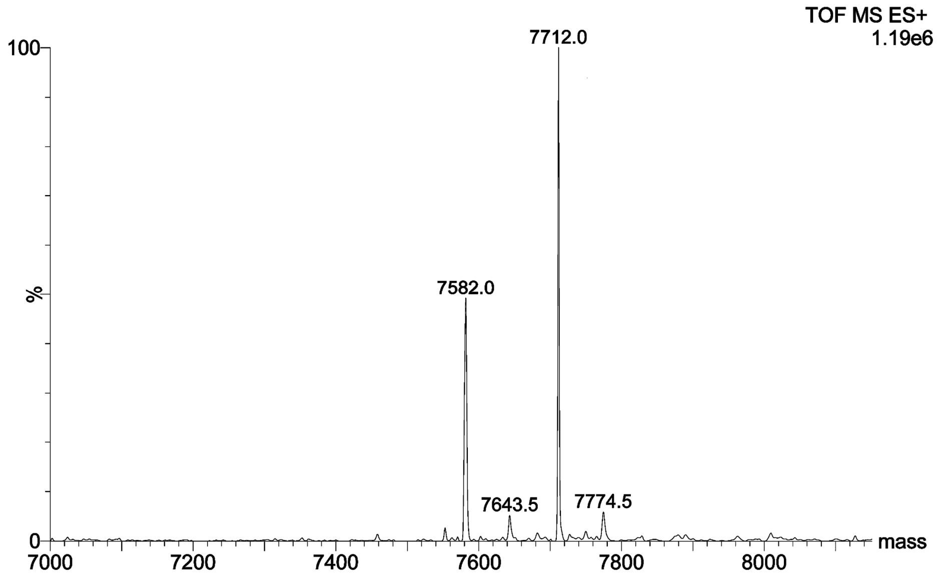

Figure S2. Deconvoluted ESI-MS spectrum of Zn_6E_c-1 after addition of 1 equiv. of Cu(I) acquired at pH 2. The masses of 7712.0 Da and 7582.0 Da correspond to apo-E_c-1 with and without the N-terminal Met residue (M(calc) 7712.5 Da and 7581.4 Da), respectively, and show that the protein is in the fully reduced state. The additional signals at 7643.5 and 7774.5 Da can be assigned to the two E_c-1 species in complex with one Cu(I) ion each and after deprotonation of two thiolate groups required for coordination (M(calc) 7775.0 Da and 7643.9 Da). Residual Cu(I) coordination is due to the rather low apparent pK_a value of the Cys thiolates in presence of Cu(I), *i.e.*, around pK_a ~ 2 [37,38]). However, the exact apparent pK_a values for the mixed Zn(II)Cu(I)-E_c-1 species were not determined and in addition, the different charges of the apo- and Cu_1-form do not allow a precise quantification of species.

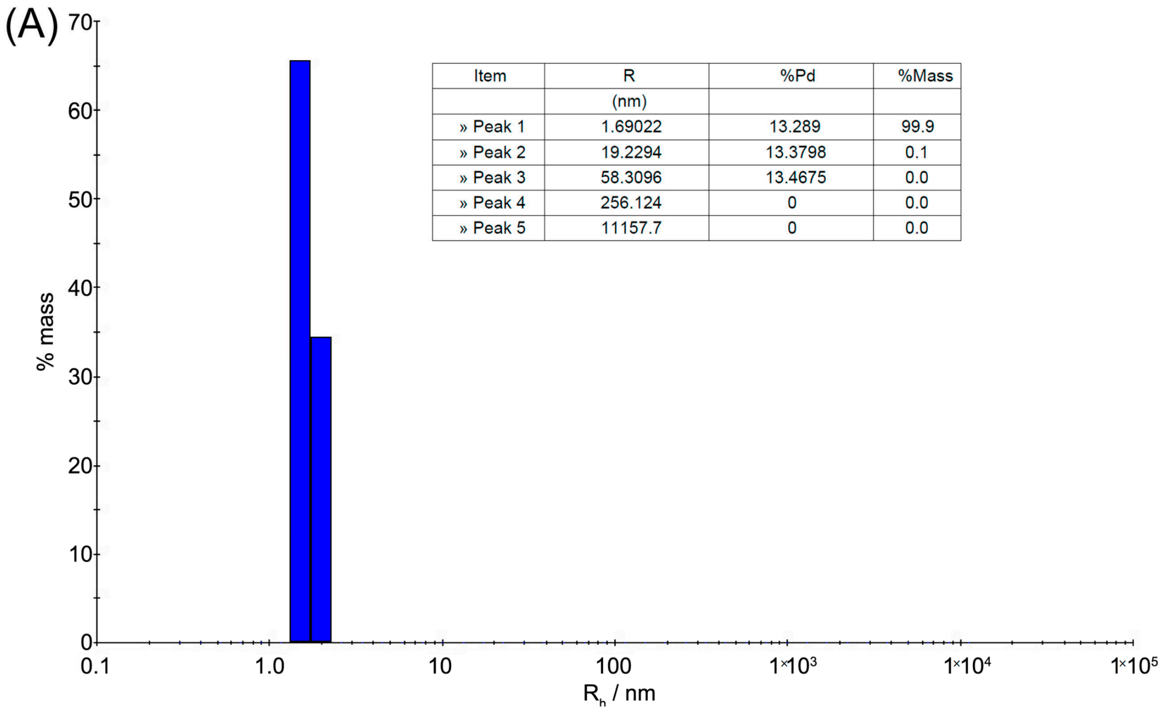

Item	R	%Pd	%Mass
	(nm)		
» Peak 1	1.69022	13.289	99.9
» Peak 2	19.2294	13.3798	0.1
» Peak 3	58.3096	13.4675	0.0
» Peak 4	256.124	0	0.0
» Peak 5	11157.7	0	0.0

Figure S3. *Cont.*

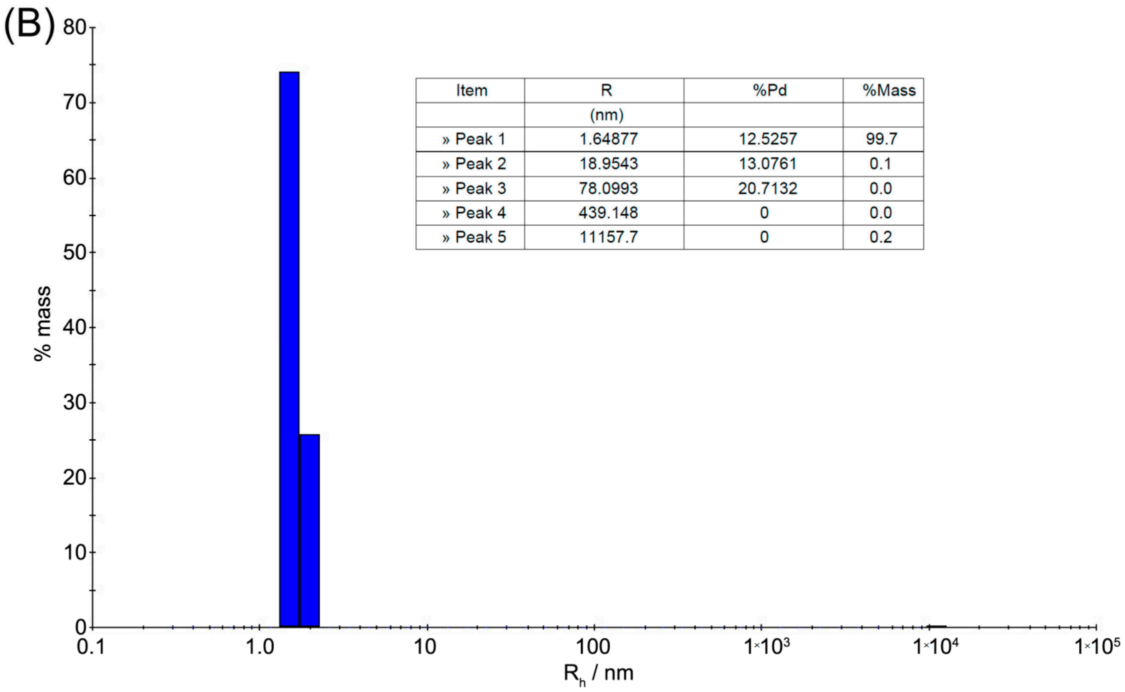

Item	R (nm)	%Pd	%Mass
» Peak 1	1.64877	12.5257	99.7
» Peak 2	18.9543	13.0761	0.1
» Peak 3	78.0993	20.7132	0.0
» Peak 4	439.148	0	0.0
» Peak 5	11157.7	0	0.2

Figure S3. Examples of histograms from dynamic light scattering experiments conducted with (**A**) Zn_6E_c-1 and (**B**) $CuZn_5E_c$-1 revealing identical hydrodynamic radii of the two protein forms, *i.e.*, 1.69 and 1.65 nm for the depicted measurements, and only very minor traces of higher molecular mass species such as, e.g., E_c-1 aggregates (buffer: 10 mM Tris-HCl, 10 mM NaCl, pH 7.5; temperature: 15 °C; number of acquisitions: 10).

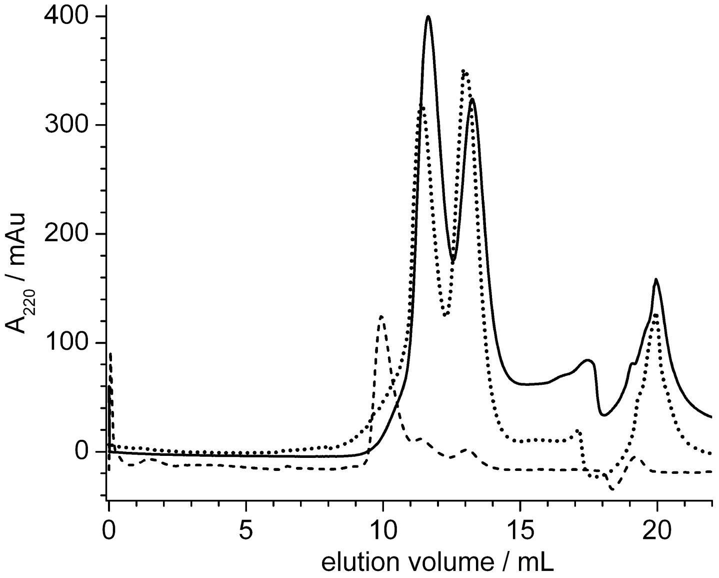

Figure S4. Size exclusion chromatogram of full-length Zn_6E_c-1 (dashed line) as well as Zn_6E_c-1 (solid line) and $CuZn_5E_c$-1 (dotted line) after digestion with proteinase K. The peak at 10.0 mL corresponds to full-length Zn_6E_c-1, at approx. 11.5 mL to the β_E-, and at approx. 13.2 mL to the γ-domain of E_c-1. (column: HiLoad 16/600 Superdex 75 pg column (GE Healthcare, Glattbrugg, Switzerland); buffer: 10 mM ammonium acetate, pH 7.5). Additional peaks above approximately 17 mL belong to small molecular weight components of the buffer.

(A)

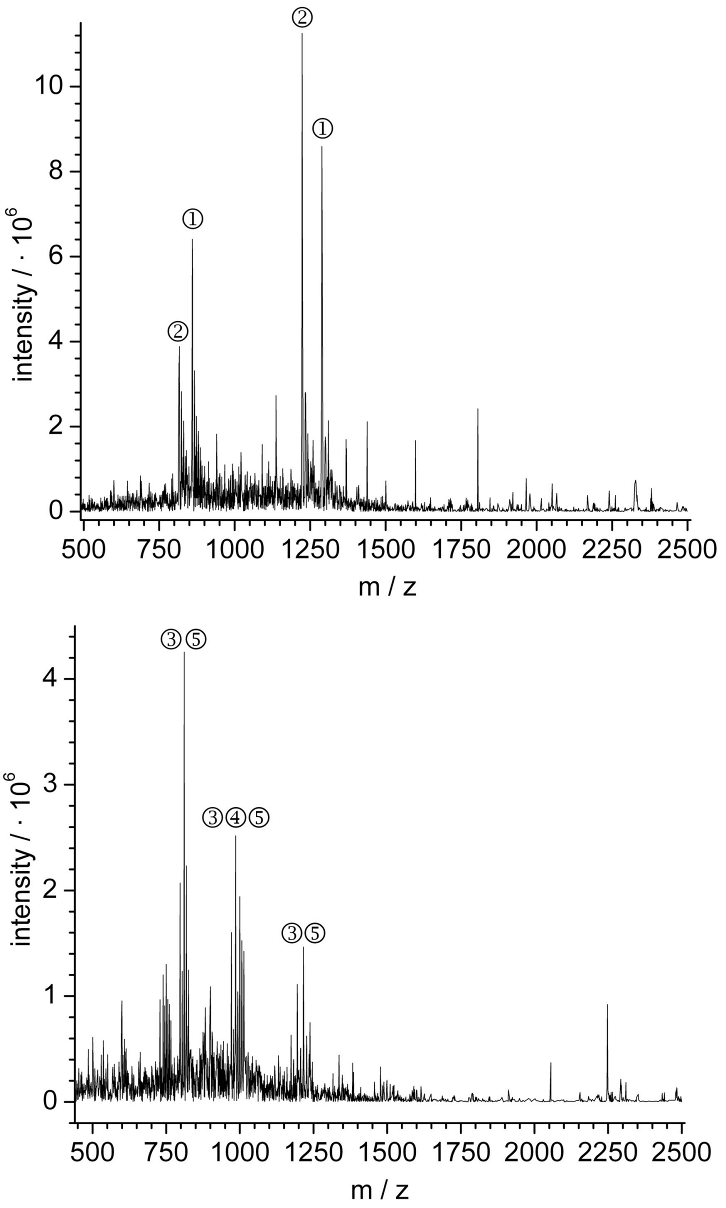

(C)

```
  MGCDDKCGCAVPCPGGTGCRCTSARSGAAAGEHTTCGCGEHCGCNPCACGREGTPSGRANRRANCSCGAACNCASCGSATA

① MGCDDKCGCAVPCPGGTGCRCTSAR
②  GCDDKCGCAVPCPGGTGCRCTSAR

③                       RSGAAAGEHTTCGCGEHCGCNPCACGREGTPSGRANRRANCSCGAACNCA
④                             GEHTTCGCGEHCGCNPCACGREGTPSGRANRRANCSCGAACNCASCGSATA
⑤                            AGEHTTCGCGEHCGCNPCACGREGTPSGRANRRANCSCGAACNCASCGSA
```

Figure S5. Analysis of peptide fragments obtained after digestion of Zn_6E_c-1 with proteinase K. The non-deconvoluted ESI-MS spectrum of the peak at 13.2 mL (Figure S5) is shown in (**A**) and main signals are assigned to the respective fragments of the γ-E_c-1 domain as shown in part (**C**) and Table S2 (below); (**B**) Non-deconvoluted ESI-MS spectrum of the peak at 11.5 mL (Figure S5) revealing the fragments of the β_E-domain shown in (**C**) as main signals. The spectra were measured in ammonium acetate buffer, pH 7.5, and hence also various Zn(II)-adducts can be detected. However, Zn(II)-binding to the β_E-domain is not stable under the ionization conditions used and hence only adducts with 1 or 2 Zn(II) ions but not more are detected.

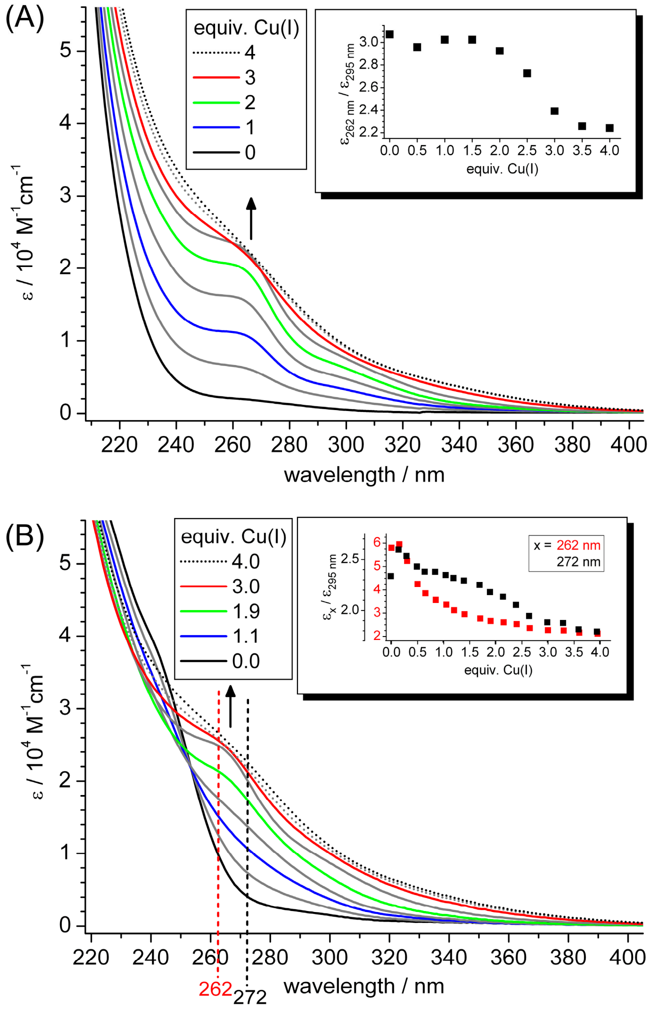

Figure S6. Set of UV/VIS spectra of the titration of apo-γ-E_c-1 (**A**) and Cd_2γ-E_c-1 (**B**) with Cu(I) ions. Dotted spectra indicate the point of constant spectral features. Insets: Absorptivity ratio $\varepsilon_{262\,nm}/\varepsilon_{295\,nm}$ to visualize the changing contribution of LMCT bands versus cluster centered transitions. Due to the strong overlap of S → Cd LMCT bands around 250 nm with S → Cu transitions also the ratio $\varepsilon_{272\,nm}/\varepsilon_{295\,nm}$ was plotted in the inset of (**B**).

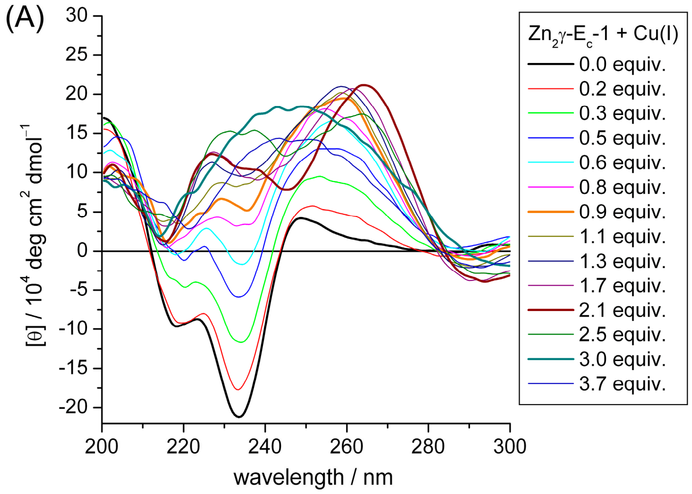

Figure S6. *Cont.*

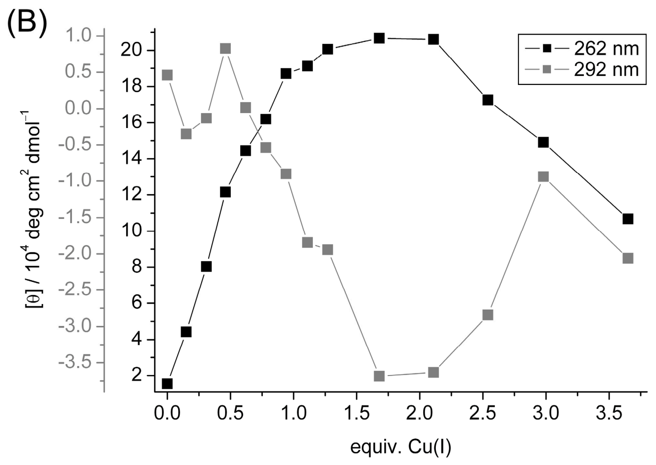

Figure S7. Titration of $Zn_2\gamma$-E_c-1 with Cu(I) ions followed by CD spectroscopy. (**A**) Evolution of CD spectra; (**B**) Course of ellipticity values at, or close to, the wavelength of the newly developing maxima observed in the CD spectra.

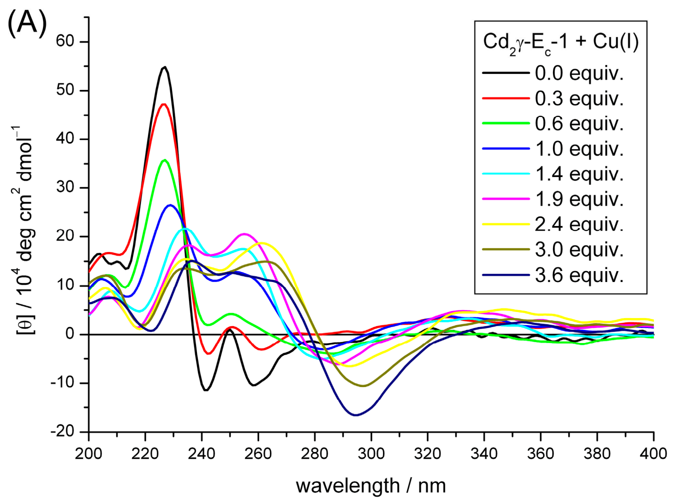

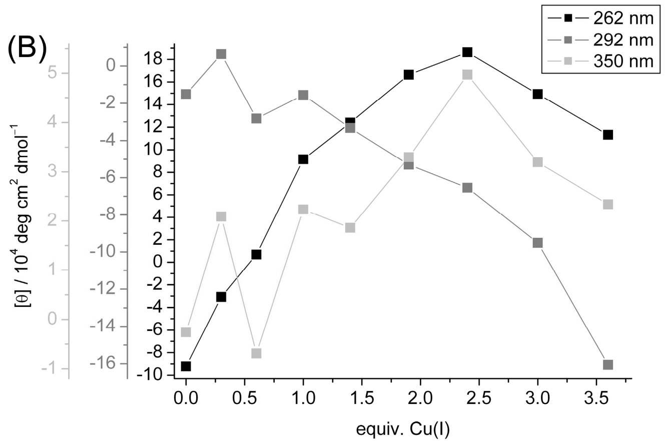

Figure S8. Titration of $Cd_2\gamma$-E_c-1 with Cu(I) ions followed by CD spectroscopy. (**A**) Evolution of CD spectra; (**B**) Course of ellipticity values at or close to the wavelength of the newly developing maxima observed in the CD spectra.

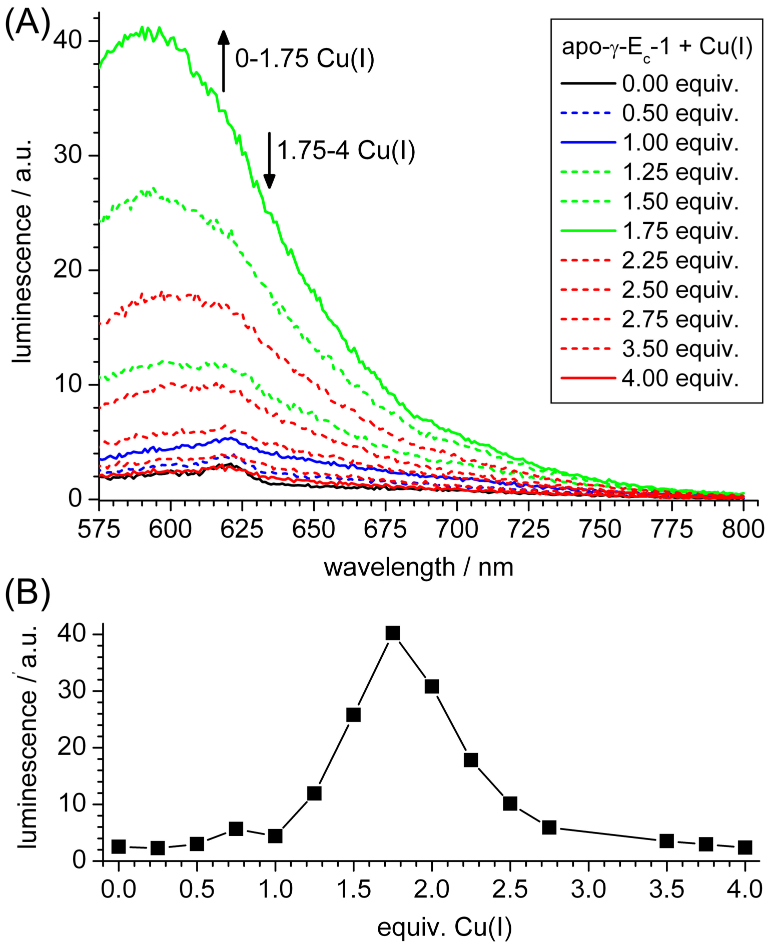

Figure S9. Dependence of room temperature luminescence at 600 nm after excitation at 280 nm from the amount of Cu(I) added to apo-γ-E_c-1. (**A**) Luminescence spectra; (**B**) plot of luminescence at 600 nm against the equiv. of Cu(I) added.

Table S1. Determination of metal ion-to-protein stoichiometries of the mixed Cu(I)-Zn(II) form of E_c-1 after addition of one equiv. of Cu(I) to Zn_6E_c-1 in 50 mM Tris-HCl, pH 7.5, and removal of loosely-bound metal ions with SEC or Chelex® 100 resin.

	**SEC**	**Chelex® 100**
[−SH]/µM (2-PDS assay before Cu(I) addition)	–	437.8
[−SH]/µM (2-PDS assay after Cu(I) addition)	308.0	412.9
[E_c-1] [a]/µM	19.25	25.81
[Zn]/µM [b]	95.29	136.00
[Cu]/µM [b]	9.44	19.44
Zn:E_c-1	4.95	5.27
Cu:E_c-1	0.7	0.75

[a] The comparison of the -SH concentration determined with the 2-PDS assay before Cu(I) addition with the value after Cu(I) addition and Chelex® 100 treatment reveals a decrease of approximately 6% (note that Chelex® 100 treatment alone does not decrease the protein concentration !) corresponding to 16 instead of 17 accessible Cys residues. This is probably due to Cu(I)-Cys coordination, which is stable at pH 4. Therefore, protein concentrations were adjusted accordingly (both for SEC and Chelex® 100). Importantly, however, no Cys disulfide formation was observed in the ESI-MS spectra excluding oxidation of thiol groups (Figure S2); [b] Metal ion concentrations were determined by F-AAS.

Table S2. Assignment of peptide fragments observed in the ESI-MS spectra (Figure S5) after digestion of Zn_6E_c-1 with proteinase K. Masses are given in Da. The circled numbers in column 1 correspond to the respective peptide fragments in Figure S5.

Species		Mass (calc)	*m*/*z* (obs), Mass (obs)				
			(+2)	(+3)	(+4)	(+5)	(+6)
①	+ 2 Zn(II)	2575.6	1288.7, 2575.4	859.5, 2575.5			
②	+ 2 Zn(II)	2444.4	1223.1, 2444.2	815.5, 2443.5			
③	+ 0 Zn(II)	4860.3			1216.0, 4860.0		811.1, 4860.6
	+ 1 Zn(II)	4923.7				985.7, 4923.5	
	+ 2 Zn(II)	4987.1				998.3, 4986.5	
④	+ 0 Zn(II)	4924.4				985.7, 4923.5	
	+ 1 Zn(II)	4987.8				998.3, 4986.5	
⑤	+ 0 Zn(II)	4823.2			1205.9, 4819.6		804.4, 4820.4
	+ 1 Zn(II)	4886.6				978.9, 4889.5	
	+ 2 Zn(II)	4950.0			1238.1, 4948.4		825.8, 4948.8

calc: calculated; obs: observed.
